# Retraction: Preliminary Evidence From an Original Study Suggests Combustion, Not Nicotine, Drives Risk and Complications of Graves' Orbitopathy

**DOI:** 10.7759/cureus.r211

**Published:** 2025-12-11

**Authors:** Matteo Acanfora, Alberto Vassallo

**Affiliations:** 1 Institute of Endocrine and Metabolic Sciences, Istituto di Ricovero e Cura a Carattere Scientifico (IRCCS) Ospedale San Raffaele, Milan, ITA; 2 Endocrinology Unit, Vita-Salute San Raffaele University, Milan, ITA; 3 Institute of Endocrine and Metabolic Sciences, Institute of Endocrine and Metabolic Sciences, Istituto di Ricovero e Cura a Carattere Scientifico (IRCCS) Ospedale San Raffaele, Milan, ITA

This article has been retracted due to the presence of critically small subgroup sample sizes that render the statistical analyses underpowered. The authors' conclusions regarding reduced or absent risk of Graves' orbitopathy in non-combustion nicotine users are unsupported by the available data and substantially overstated. As a result, the findings are not reliable and should not be used to guide clinical interpretation or harm reduction counseling. In addition, the manuscript was submitted to the journal without formal authorization from the affiliated hospital institution, constituting a breach of institutional policies governing data ownership, authorship, and publication rights, necessitating the removal of the article contents at the time of retraction.

